# Phacoemulsification Versus Manual Small Incision Cataract Surgery: An Umbrella Review of Systematic Reviews

**DOI:** 10.7759/cureus.96753

**Published:** 2025-11-13

**Authors:** Osman Haji, Ling Paulina Gronczewska, Alexander S Green, Sirjhun Patel

**Affiliations:** 1 General Practice, King’s College Hospital NHS Foundation Trust, London, GBR; 2 General Medicine, West Suffolk Hospital, Bury St Edmunds, GBR; 3 Ophthalmology, West Suffolk Hospital, Bury St Edmunds, GBR

**Keywords:** best corrected visual acuity (bcva), cataract, manual small‑incision cataract surgery, phacoemulsification surgery, uncorrected distance visual acuity

## Abstract

Phacoemulsification and manual small incision cataract surgery (MSICS) are two widely practised techniques for cataract surgery worldwide. However, their comparative efficacy in terms of postoperative visual acuity (VA) remains debated. This paper is an umbrella review for this topic, which synthesises evidence from systematic reviews and meta-analyses directly comparing VA outcomes between the two techniques.

PubMed, Cochrane, Web of Science, and the Excerpta Medica database (Embase) were searched, with 712 results remaining after de-duplication. After screening, six papers underwent full-text review, leaving four systematic reviews/meta-analyses for final inclusion. Inclusion criteria were English-language systematic reviews directly comparing postoperative VA between phacoemulsification and MSICS in adults undergoing cataract surgery. Data extraction focused on uncorrected and best-corrected VA (BCVA) at early (≤1 week), intermediate (six to eight weeks), and late (≥6 months) time points. Methodological quality was assessed using A Measurement Tool to Assess Systematic Reviews 2 (AMSTAR 2).

The four included reviews ranged from six to 11 primary studies each, with total sample sizes ranging from 1315 to 76,838 eyes. They were published in the years 2013 and 2015. Across reviews, no statistically significant differences were consistently found in BCVA at any time point. Two reviews reported small early advantages for phacoemulsification in uncorrected VA (UCVA, odds ratios (ORs) of 1.40 and relative risk (RR) of 0.90 for achieving ≥6/9 or ≥6/18, respectively), but these differences were not maintained at later follow-up. Complication profiles were broadly similar. AMSTAR 2 ratings indicated high confidence in the Cochrane review, moderate confidence in two reviews, and low-to-moderate confidence in one review.

Evidence from conducted systematic reviews suggests that phacoemulsification and MSICS yield similar postoperative VA outcomes. As such, the choice of technique may be guided more by resource availability, surgeon expertise, and patient-specific considerations.

## Introduction and background

Cataract remains the leading cause of blindness worldwide, responsible for approximately 50% of global blindness and a significant proportion of visual impairment across all age groups [1]. Currently, an estimated 94 million people aged 50 years and over have moderate-to-severe distance vision impairment or blindness that could be corrected through access to cataract surgery [2]. The global burden is disproportionately concentrated in low- and middle-income countries (LMICs) [3], where access to surgical services is often limited and patients tend to present with advanced lens opacities [4]. Cataract surgery is one of the most cost-effective medical interventions, consistently ranked high in disability-adjusted life years (DALYs) averted per unit cost [5]. Consequently, improving surgical capacity and efficiency has been a major focus of global ophthalmic public health strategies, including the World Health Organization’s “Vision 2020: The Right to Sight” initiative [6].

Phacoemulsification and manual small incision cataract surgery (MSICS) are two of the most widely practised examples of cataract surgery techniques. Each has distinct technical, logistical, and economic implications, and their relative merits continue to be debated in both academic literature and clinical practice.

Phacoemulsification employs ultrasonic energy to fragment the opacified crystalline lens, which is then aspirated through a small corneal incision, typically 2.2-3.0 mm in width, with a foldable intraocular lens implanted through the same incision [7]. The small wound size confers several theoretical advantages: reduced surgically induced astigmatism (SIA), faster refractive stabilisation, reduced wound leakage risk, and often sutureless closure [7]. Phacoemulsification is regarded as the standard of care in most high-income countries [8], with evidence supporting rapid postoperative visual recovery, good long-term visual outcomes, and compatibility with smaller, foldable lenses [9]. However, compared to other techniques, it requires greater investment in and maintenance of equipment [10]. It also demands a higher level of technical skill and perhaps a longer learning curve for surgeons [11].

MSICS evolved from another surgical technique called extracapsular cataract extraction (ECCE) and involves creating a self-sealing scleral tunnel incision, up to 9 mm in size, through which the entire nucleus is delivered manually without the use of ultrasound, with an IOL being implanted into the capsular bag thereafter [12]. MSICS avoids the need for costly phacoemulsification machines and associated equipment [13] and is particularly well-suited for dense, brunescent cataracts common in LMIC populations [14].

A key outcome of interest in comparing these techniques is postoperative visual acuity (VA), which may be measured as uncorrected VA (UCVA) or best-corrected VA (BCVA). UCVA reflects the patient’s immediate unaided functional vision after surgery and is often of great importance in settings where access to postoperative refractive correction is limited. BCVA, typically assessed at later time points, provides a measure of the optical potential of the operated eye once refractive errors are corrected [15]. Early postoperative UCVA may be influenced by factors such as wound size and corneal oedema, whereas BCVA in the medium to long term may better reflect the stability of refractive outcomes and the absence of visually significant complications [16,17].

Randomised controlled trials (RCTs) and observational studies comparing phacoemulsification and MSICS have reported mixed results. Some have suggested that one technique may have better postoperative visual acuity outcomes, whereas many others have reported no statistically significant differences. Differences in study design, sample size, patient population, surgical technique, and outcome reporting make it challenging to draw definitive conclusions from individual studies alone.

To address this, a number of systematic reviews and meta-analyses have been conducted, pooling data from RCTs and, in some cases, high-quality observational studies. These reviews vary considerably in scope and quality. Differences in inclusion criteria, follow-up intervals, outcome definitions, and statistical methods have led to variations in reported findings. Moreover, not all reviews adhere to best-practice methodological standards such as protocol registration, comprehensive search strategies, dual independent screening, and assessment of publication bias.

When multiple systematic reviews on the same question exist, it can be difficult for clinicians and policymakers to identify the most reliable synthesis. An umbrella review, a systematic review of systematic reviews [18], addresses this problem by collating and critically appraising all relevant systematic reviews and meta-analyses on a given topic [19]. Using tools such as A Measurement Tool to Assess Systematic Reviews 2 (AMSTAR 2) [20], umbrella reviews can evaluate the methodological quality of each included review, identify areas of consensus, and provide a comprehensive overview of research conducted in answering a specific question [21].

Given that both phacoemulsification and MSICS are widely practised worldwide, and that surgical decision-making often involves trade-offs between clinical outcomes, resource constraints, and staff training considerations, an umbrella review comparing their postoperative VA outcomes may go some way in identifying which method is better in given contexts. Such a review not only has implications for high-income countries seeking to optimise patient satisfaction and refractive outcomes, but also for LMICs where policymakers will need to be more cognisant of sustainability and cost-effectiveness.

This umbrella review aims to synthesise evidence from published systematic reviews and meta-analyses directly comparing postoperative VA outcomes between phacoemulsification and MSICS in adult cataract patients. The primary objective is to determine whether one technique provides superior postoperative visual acuity at early (≤1 week), intermediate (six to eight weeks), or late (≥6 months) follow-up. Secondary objectives include summarising the risk of complications from each technique where reported. By also appraising the methodological rigour of included reviews, this study seeks to inform surgical practice guidelines, training priorities, and health policy decisions in diverse global contexts.

## Review

Methods

Study Design

This umbrella review was conducted following the Preferred Reporting Items for Systematic Reviews and Meta-Analyses (PRISMA) 2020 guidelines for overviews of reviews. The objective was to synthesise and appraise evidence from published systematic reviews and meta-analyses comparing postoperative visual acuity outcomes between phacoemulsification and MSICS in adult patients undergoing cataract surgery.

Eligibility Criteria

We included systematic reviews and meta-analyses of RCTs or high-quality observational studies that directly compared phacoemulsification and MSICS, reported postoperative VA (uncorrected or best corrected) as a primary or secondary outcome, and were published in English. Reviews were excluded if they were narrative reviews or expert opinions/commentaries, included combined procedures (e.g., cataract surgery with trabeculectomy), did not report VA outcomes separately for each surgical technique, or were published in languages other than English.

Search Strategy

A comprehensive search was conducted in April 2025 in the following databases: PubMed, Excerpta Medica database (Embase), Cochrane Library, and Web of Science. Search terms included controlled vocabulary and synonyms for cataract surgery, phacoemulsification, and MSICS (Appendix A). Equivalent strategies were adapted for Embase, Cochrane Library, and Web of Science using database-specific syntax. No date restrictions were applied.

Study Selection

All retrieved records were imported into reference management software (Zotero, Corporation for Digital Scholarship, Vienna, VA), and duplicates were removed. Two reviewers independently screened titles and abstracts against the eligibility criteria. Full texts were obtained for potentially relevant studies and assessed in duplicate for inclusion, with disagreements resolved by discussion.

Data Extraction

From each included review, data were extracted on author, year of publication, and country of corresponding author; number and type of included primary studies; total sample size and follow-up duration; postoperative UCVA and BCVA outcomes at early (≤1 week), intermediate (six to eight weeks), and late (≥6 months) time points; secondary outcomes such as SIA, refractive error, and complication rates; summary effect estimates and statistical significance; and methodological quality rating (AMSTAR 2).

Quality Assessment

The methodological quality of each included systematic review was assessed using the AMSTAR 2 checklist, which evaluates 16 domains, including protocol registration, comprehensiveness of the literature search, duplicate study selection and data extraction, risk of bias assessment for included studies, and evaluation of publication bias. AMSTAR 2 overall confidence ratings (high, moderate, low, or critically low) were subsequently assigned. This was done by two reviewers, with disagreements resolved by discussion between the two reviewers.

Synthesis of Results

Due to the nature of an umbrella review, no de novo meta-analysis of primary studies was performed. Instead, the pooled effect estimates and conclusions from the included systematic reviews/meta-analyses were narratively synthesised, with attention to areas of agreement, conflict, and methodological strengths or weaknesses.

Study Selection 

**Figure 1:**
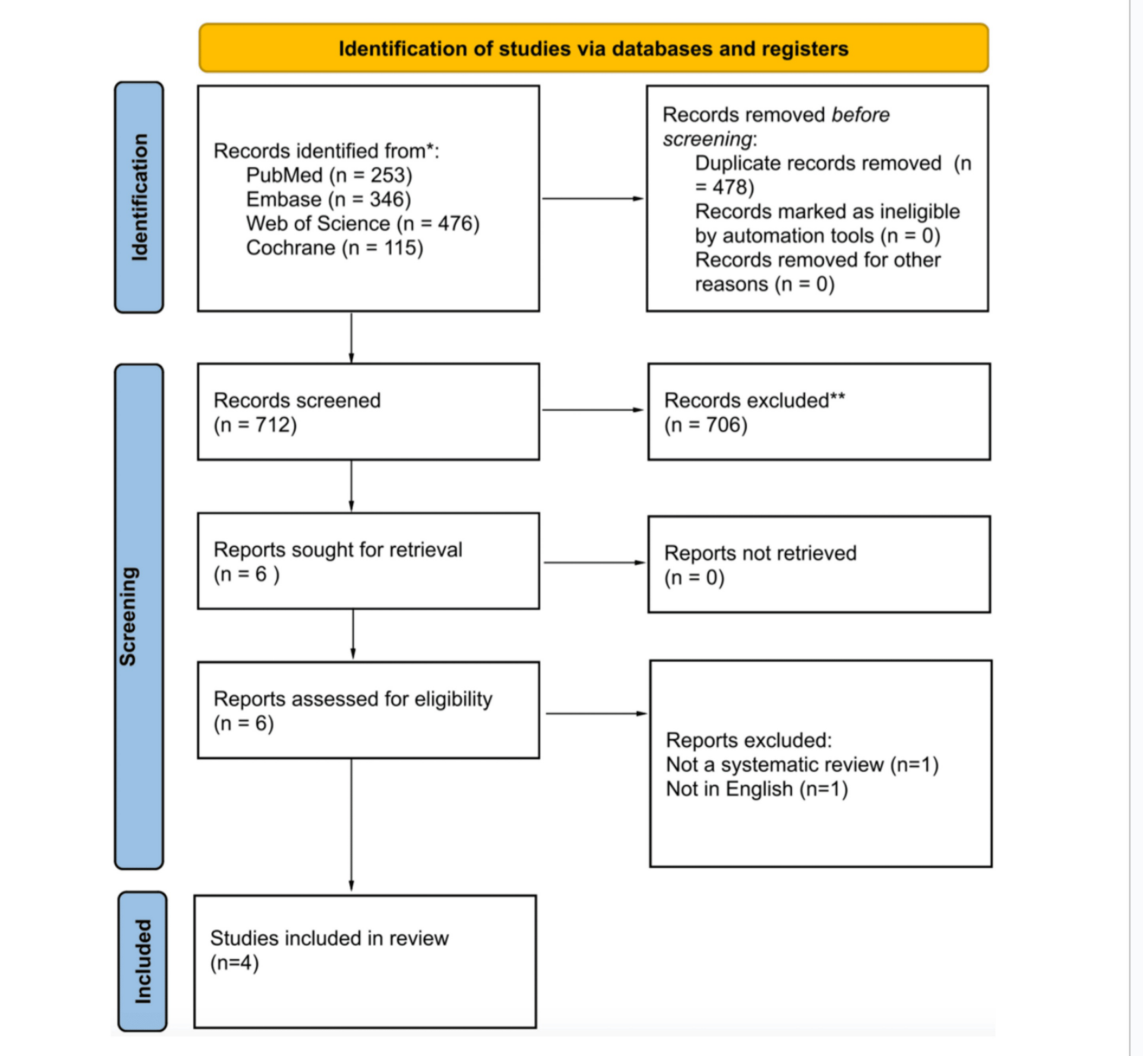
A PRISMA flowchart outlining the study selection process PRISMA: Preferred Reporting Items for Systematic Reviews and Meta-Analyses; Embase: Excerpta Medica database

The search yielded 1,190 records: PubMed (n = 253), Embase (n = 346), Web of Science (n = 476), and Cochrane Library (n = 115). After removal of duplicates, 712 unique records were screened by title and abstract. Of these, 706 were excluded, leaving six articles for full-text review. Two were then excluded (one was a non-English language review, and the other was found to be a narrative literature/scoping review rather than a true systematic review). Four systematic reviews met the inclusion criteria and were included in the final synthesis. This can be seen in the PRISMA flow diagram in Figure 1.

Results

Overview of Included Reviews

Four systematic reviews and meta-analyses met the inclusion criteria for this umbrella review. They were published between 2013 and 2015, conducted in diverse geographic and economic contexts, and included between six and 11 papers each. All reviews compared postoperative VA outcomes between phacoemulsification and MSICS, albeit with differences in the length of follow-up and presentation of the data.

A summary of key characteristics for each review is presented in Table 1, and their AMSTAR 2 methodological quality ratings are shown in Table 2.

**Table 1:** Summary of the key characteristics for each review CENTRAL: Cochrane Central Register of Controlled Trials; MEDLINE: Medical Literature Analysis and Retrieval System Online; Embase: Excerpta Medica database; LILACS: Latin American and Caribbean Literature on Health Sciences; CPCI-S: Conference Proceedings Citation Index – Science; mRCT: metaRegister of Controlled Trials; ICTRP: International Clinical Trials Registry Platform; MSICS: manual small incision cataract surgery; PCIOL: posterior chamber intraocular lens; RCTs: randomized controlled trials; pres. VA: presenting visual acuity; BCVA:  best-corrected visual acuity; UCVA: uncorrected visual acuity; RR: relative risk; OR: odds ratio; Cochrane RoB: Cochrane Risk of Bias tool; PE: phacoemulsification; SIA: surgically induced astigmatism; ECC: endothelial cell count; NS: not significant; Intraop comps: intraoperative complications; Postop comps: postoperative complications; SICS: small incision cataract surgery; OCTET: Oxford Cataract Treatment and Evaluation Team; CBM: Chinese Biomedical Literature Database; CNKI: Chinese National Knowledge Infrastructure

Citation	Study types included	Databases & last search	Eligibility	Studies / Eyes	Follow-up window	Primary outcomes	Key quantitative findings	Complications	Cost	Risk of bias tool
Riaz et al., 2013 [22]	RCTs	CENTRAL, OVID MEDLINE (incl. OVID MEDLINE In-Process), Embase, LILACS, Web of Science CPCI-S, mRCT, ClinicalTrials.gov, WHO ICTRP; searched up to 23 July 2013	Age-related cataract; MSICS vs. PE with PCIOL; RCTs only	8 RCTs; 1708 participants	One day–six months (mostly six–eight weeks)	Good functional vision (pres. VA 6/12+); Poor outcome (BCVA <6/60). Presenting VA not reported; BCVA/UCVA used	Seven studies reported BCVA ≥6/18 at six to eight weeks: RR 0.99 (0.98–1.01), no difference. Three studies report UCVA ≥6/18 at six to eight weeks: RR 0.90 (0.84–0.96) →favouring PE. One trial reported UCVA at six months: RR 1.07 (0.91–1.26), no diff. BCVA <6/60: Peto OR 2.48 (0.74–8.28), favours PE but uncertain.	Low numbers, the review was underpowered to detect differences	PE>4× cost of MSICS (Ruit 2007)	Cochrane RoB
Zhang et al., 2013 [23]	RCTs	Cochrane Library, PubMed, Embase; up to 1 Jan 2012	RCTs; age-related cataract; MSICS vs. PE; PCIOL; ≥6 weeks follow‑up; no other ocular disease	6 RCTs; 1315 eyes	Six weeks–six months	Proportion with good visual outcome (BCVA ≥6/9), or poor visual outcome (BCVA <6/18); UCVA ≥6/9; UCVA <6/18 also measured	BCVA 6/9+: No significant difference (P=0.69). BCVA <6/18: No significant difference (P=0.68). UCVA 6/9+: favours PE (OR 1.40, 95% CI 1.03–1.91; P=0.03). UCVA <6/18: more in MSICS (P=0.03).	No significant differences	Not assessed	Jadad
Gogate et al., 2015 [24]	Comparative studies	PubMed, Cochrane, Scopus; plus non‑English/grey literature (dates not stated)	Comparative studies of MSICS vs. PE; outcomes at cut-offs 6/9, 6/18, 6/60; astigmatism; ECC; complications; time; cost; near UCVA	11 studies; 76,838 eyes (complication datasets); nine studies (n=1768) for many VA outcomes	Typically ~6±2 weeks; some six months	UCVA & BCVA at 6/9, 6/18, 6/60; SIA; ECC; complications; duration; cost; near UCVA	BCVA & UCVA at 6/18 and 6/60: no diff; BCVA 6/9: no diff; UCVA 6/9: favours PE (P=0.040). SIA was higher with SICS (P=0.005). ECC loss: NS (P=0.298). Intraop comps: NS (P=0.964); Postop comps: NS (P=0.362). SICS is safer in the learning phase (postop comps P=0.003).	Comparable overall, learning-curve signal in favour of SICS safety	SICS < ½ cost of PE; SICS quicker	OCTET grading for complications; diverse designs
Ye et al., 2015 [25]	Prospective, randomised study	Medline, PubMed, CBM, CNKI (dates not stated)	Prospective randomised clinical trials comparing MSICS vs. PE; age‑related cataract	10 trials (per authors)	One to 12 weeks in the table; some to 12 weeks	UCVA at one week; posterior capsular rupture; day 1 corneal oedema	One‑week UCVA: OR 0.84 (0.67–1.06), P=0.15 (NS). Posterior capsule rupture: OR 1.07 (0.73–1.58), P=0.72 (NS). Day‑1 corneal oedema: OR 0.90 (0.70–1.16), P=0.42 (NS).	No significant differences across reported outcomes	Not assessed	Jadad scoring; fixed‑effects models predominantly

**Table 2:** AMSTAR 2 methodological quality ratings for each review RoB: risk of bias; COI: conflict of interest; RCTs: randomized controlled trials; RR: relative risk; OR: odds ratio; Embase: Excerpta Medica database; WMD: weighted mean difference; OCTET: Oxford Cataract Treatment and Evaluation Team; CBM: Chinese Biomedical Literature Database; CNKI: Chinese National Knowledge Infrastructure; PRISMA: Preferred Reporting Items for Systematic Reviews and Meta-Analyses; AMSTAR 2: A Measurement Tool to Assess Systematic Reviews 2

Review	Protocol registered?	Comprehensive search	Justification of study designs	Duplicate selection/data extraction	RoB assessment	List of excluded studies	Meta-analysis approach	Heterogeneity explored	Publication bias assessed	Funding/COI of included studies	Overall appraisal
Riaz et al., 2013 [22]	Cochrane protocol published	Yes (multi‑db incl. trial registries)	Yes (RCTs only)	Yes (two authors)	Cochrane tool; reported	Likely (standard Cochrane tables)	RR/OR; fixed/random as appropriate	Yes (I², random‑effects if needed)	Not enough studies to assess formally	Not clearly reported	High methodological quality; underpowered outcomes
Zhang et al., (2013) [23]	Unclear	Yes (Cochrane, PubMed, Embase)	RCTs only	Yes (two reviewers; third to resolve)	Jadad + additional items	Partially described	OR/WMD; fixed unless I²>50% then random	Yes (χ², I²)	Yes (Begg/Egger)	Not stated	Moderate quality; limited by short follow‑up
Gogate et al., (2015)[24]	Unclear	Yes (PubMed, Cochrane, Scopus, non‑English, grey)	Included RCTs + other comparative studies	Not explicit	OCTET grading for complications; limited formal RoB reporting	Narratively described	Random‑effects (DerSimonian‑Laird)	Yes (χ², I²)	Not clearly reported	Not stated	Moderate (broader scope; heterogeneous designs)
Ye et al., (2015) [25]	Unclear	Yes (Medline, PubMed, CBM, CNKI + manual)	Prospective randomised trials only	Yes (two reviewers)	Jadad scoring (many low scores)	Not detailed; PRISMA figure provided	Fixed‑effects predominantly	Q test; subgroup if needed	Funnel plot shown	Not stated	Low‑to‑moderate (short follow‑up; low Jadad; narrow outcomes)

Review 1 

Riaz et al.'s study (2013) [22] was a Cochrane systematic review of eight RCTs (n = 1708; India, Nepal, South Africa) that compared MSICS with phacoemulsification for age-related cataract, with follow-up mostly at six to eight weeks and up to six months in two trials. Trials were generally at risk of bias because of unclear masking and incomplete follow-up; evidence quality for many outcomes was graded low/very low.

VA outcomes measured included UCVA ≥6/18 (≈ good functional vision proxy) at six to eight weeks. The data were taken from three RCTs with a total sample size of 767, and the results favoured phacoemulsification (pooled relative risk (RR) 0.90, 95% CI 0.84-0.96). As per one RCT, at six months, there was no significant difference: RR 1.07 (0.91-1.26). The second recorded outcome was a BCVA ≥6/18 at six to eight weeks. Results extracted from seven RCTs with a total of 1223 patients suggested no difference, with a pooled RR of 0.99 (0.98-1.01). The third recorded measure was a poor outcome, defined as a BCVA <6/60 ≤3 months. Such events were rare (8/617 MSICS vs 3/606 phacoemulsification, with a pooled Peto odds ratio (OR) of 2.48 (0.74-8.28)), suggesting a large degree of uncertainty.

Complications: The review also recorded outcomes with regard to complications. For example, posterior capsule rupture. In this, there was no clear difference, as Peto OR was 1.07 (0.63-1.83). With regard to iridodialysis, there were very few events (7). The Peto OR was 2.37 (0.54-10.45), meaning results here would be obscured by substantial uncertainty. Capsulorrhexis extension occurred numerically more frequently with phacoemulsification, with a Peto OR of 0.26 (0.05-1.30). Early postoperative corneal oedema (day 1-7) was higher with phacoemulsification. The pooled Peto OR was 0.58 (0.41-0.83) (OR <1 favours MSICS for fewer oedema events). Posterior capsule opacification (occurring ≤6 months) was higher with MSICS in one RCT (20/46 MSICS vs 7/48 phacoemulsification), with an RR of 2.98 (1.39-6.37).

Other outcomes: With endothelial cell loss, there was no significant difference overall (e.g., six-week loss of ~5.4% phacoemulsification vs. 4.2% MSICS in one RCT; another RCT: 18.4% vs. 17.7% at six weeks). Surgically induced astigmatism, at six to eight weeks, was greater with MSICS in several trials, but by six months, there was no significant difference. Conversions from phacoemulsification to MSICS were reported (e.g., 2/199, 5/100, 8/100, 3/137) for hard nuclei or complications. Note was also made of surgical times and costs: MSICS was faster and cheaper (e.g., ~9 minutes and USD 15 for MSICS vs. ~15.5 minutes and USD 70 for phacoemulsification in one RCT).

AMSTAR assessment: Strengths included that the protocol was prospectively registered; the search was comprehensive (multiple databases + trial registries); study selection and data extraction were conducted in duplicate; and the risk of bias was assessed using the Cochrane tool. Heterogeneity was explored appropriately, and effect measures were clearly reported. Limitations included that not enough trials were available to allow a formal assessment of publication bias and that some outcomes were underpowered due to small sample sizes. Overall, there was high methodological quality, though the certainty of findings was constrained by limited primary data.

Synthesis for this review: Cochrane concludes phacoemulsification improves short-term UCVA, but BCVA is similar between techniques. There is also a paucity of evidence for rare adverse outcomes. MSICS offers lower cost and shorter operating times, with more early corneal oedema seen after phacoemulsification and more posterior capsule opacification reported with MSICS at six months in one RCT. These findings align with Review 1 (Gogate et al., 2015): similar BCVA, modest UCVA edge for phacoemulsification, less SIA with phacoemulsification, and cost/speed advantages for MSICS.

Review 2

Overview of the included review: Zhang et al.'s study, conducted in 2013 [23], was a meta-analysis published in Clinical & Experimental Ophthalmology. It pooled six RCTs (n = 1,315 eyes; phacoemulsification = 651, MSICS = 664) comparing phacoemulsification with MSICS for age-related cataract, with follow-up of six weeks to six months. Searches covered Cochrane, PubMed, and EMBASE to 1 Jan 2012; primary outcomes were BCVA ≥6/9 and BCVA <6/18, with UCVA thresholds, SIA, endothelial cell count (ECC) loss, and complications as secondary outcomes. Trial quality (Jadad scale) was generally good; randomisation was appropriate, and several trials used masking. Publication bias tests were not significant (Begg p=0.26, Egger p=0.12).

VA outcomes measured included BCVA ≥6/9 (end of follow-up). The review found no significant difference (three RCTs; OR 1.23, 95% CI 0.45-3.36; I²=74%). A second outcome was BCVA < 6/18 (end of follow-up). Again, no clear difference was found (all six RCTs; OR 0.77, 95% CI 0.23-2.61; I²=0%). The third outcome was UCVA ≥6/9. This favoured phacoemulsification (three RCTs; OR 1.40, 95% CI 1.03-1.91; p=0.03). A fourth recorded outcome was UCVA <6/18: more frequent with MSICS (three RCTs; pooled OR 0.65, 95% CI 0.44-0.95; p=0.03 when comparing phacoemulsification vs. MSICS).

Cornea/endothelium and astigmatism: No significant difference was found between the two techniques in endothelial cell loss (two RCTs; weighted mean difference (WMD) 0.86%, 95% CI −1.38 to 3.11; p=0.45). SIA was lower with PE (two RCTs; WMD −0.40 D, 95% CI −0.47 to −0.32; p<0.00001).

Complications: There was no clear difference in either intraoperative complications (five RCTs; RR 1.24, 95% CI 0.72-2.13; p=0.78) or postoperative complications (five RCTs; RR 0.91, 95% CI 0.29-2.88; p=0.87), though with the latter, individual trials reported more day-1 corneal oedema with WMD and more hyphaema/posterior capsular opacification with MSICS; the review pooled all adverse events without stratifying by type.

AMSTAR assessment: Strengths include a comprehensive database search, duplicate selection with adjudication, assessment of methodological quality using Jadad plus additional items, and the fact that heterogeneity and publication bias were formally tested (Begg/Egger). Limitations include that the protocol registration was not reported, the list of excluded studies was incomplete, and the use of the Jadad scale, as it provides only a limited appraisal of bias domains. Follow-up across included RCTs was also short (≤6 months). Overall, this paper was of moderate quality, with appropriate analytic methods but limited by short follow-up and reliance on Jadad scoring.

Synthesis for this review: This meta-analysis concludes similar BCVA between techniques, a modest UCVA advantage for phacoemulsification, significantly less SIA with phacoemulsification, no difference in endothelial cell loss, and no clear difference in pooled complication rates (with early corneal oedema tending to be higher after phacoemulsification and posterior capsule opacification reported higher after MSICS in single trials). These findings align with Review 1 (Gogate et al., 2015) and Review 2 (Riaz et al., 2013): comparable BCVA, slightly better uncorrected distance vision, and lower surgically induced astigmatism with phacoemulsification, and broadly similar safety profiles.

Review 3

This was a systematic review and meta-analysis (Gogate et al., 2015) [22] comparing phacoemulsification with MSICS/SICS. It searched PubMed, Cochrane, and Scopus (also hand-searching non-indexed/non-English literature) and ultimately included 11 comparative studies for complications (total n = 76,838 eyes). For visual and other outcomes, pooled analyses generally drew on subsets of these studies (excluding a large high-volume series and a residency “learning-curve” dataset), leaving nine studies (n = 1,768 eyes) for many estimates.

VA outcomes: The review found no significant difference between techniques with regard to a postoperative BCVA ≥6/18 (seven studies, 1,229 eyes; pooled OR ~0.73; 95% CI ~0.34-1.82; I² = 0%). There was also no significant difference in UCVA ≥6/18 (five studies, 1,082 eyes; pooled OR ~0.81; 95% CI ~0.51-1.29; I² ≈ 53%). As for a BCVA >6/9, again, no significant difference was noted (three studies; pooled OR ~0.81; 95% CI ~0.30-2.22). However, for UCVA >6/9, the results favoured phacoemulsification (three studies; pooled OR ~0.71; 95% CI ~0.51-0.98; p = 0.04). The paper also recorded poor outcomes, defined as <6/60. There were no significant differences for this finding (two studies; pooled OR ~0.61; 95% CI ~0.33-1.19) or in aided VA <6/60 (pooled OR ~2.19; 95% CI ~0.46-10.38).

Astigmatism and endothelial cell loss: Across seven studies (1303 eyes), phacoemulsification yielded significantly lower surgically induced astigmatism than MSICS (SMD = −0.61; 95% CI −1.05 to −0.18). The smaller incision with phacoemulsification is cited as the likely driver, though this did not consistently translate into superior uncorrected distance acuity at standard thresholds. To assess differences in endothelial cell loss, the results from two studies were pooled. However, findings were mixed, and the overall difference was not statistically significant (no clear advantage to either technique).

Intraoperative and postoperative complications: Complications were graded using Oxford Cataract Treatment and Evaluation Team (OCTET) severity weights. Excluding the high-volume and “learning-curve” datasets, no significant differences were detected between phacoemulsification and MSICS for either intraoperative or postoperative complications. When all datasets were explored in sensitivity analyses (including high-volume and trainee series), conclusions about comparative safety remained broadly similar.

Near vision (unaided): One prospective comparative study reported better unaided near VA after MSICS (e.g., N9 or better in 35% MSICS vs 3% phacoemulsification when targeted for distance emmetropia), attributed to mild against-the-rule myopic astigmatism after MSICS.

Surgery duration and costs (descriptive): A quantitative meta-analysis was not possible due to missing SDs. Reported operative times tended to be shorter for MSICS in most studies. Costs were consistently lower for MSICS in the cited program settings (e.g., India: US$42.10 (phacoemulsification) vs. US$15.34 (MSICS); Nepal: US$70 (phacoemulsification) vs. US$15 (MSICS)).

AMSTAR assessment: The paper has several strengths: there was a broad search strategy including non-English and grey literature, a large, pooled dataset for complications, and the fact that heterogeneity was explored with chi-square (χ²) and I². It did, however, have a few limitations. For example, no protocol registration was reported. The inclusion of both RCTs and observational designs may have reduced methodological consistency. Duplicate screening was also not clearly described, and the risk of bias assessment was limited (OCTET grading only). The issue of possible publication bias was also not clearly addressed. Overall, the study was of moderate quality, but conclusions were weakened by heterogeneity and a lack of systematic risk of bias assessment.

Synthesis for this review: Overall, this review found comparable visual outcomes at clinically relevant cut-offs (6/18, 6/60), a modest UCVA >6/9 advantage for phacoemulsification, less SIA with phacoemulsification, and no clear safety difference when excluding high-volume and trainee data, with MSICS showing advantages in cost, speed, and trainee safety.

Review 4

Overview of the included review: Ye et al. 2015 [25] was a meta-analysis in the International Journal of Clinical and Experimental Medicine, which compared MSICS with phacoemulsification for age-related cataract. Searches covered Medline, PubMed, CBM, and CNKI, plus manual checking. The review prespecified inclusion of prospective randomised clinical trials, assessed quality with Jadad, and used RevMan 5.0 with OR/RR and fixed/random effects based on heterogeneity. Ten studies were included, with reported follow-up ranging from one to 12 weeks. Although eligibility was restricted to RCTs, the included-study list references at least one retrospective cohort (Khanna et al., BMJ Open, 2012 [26]), indicating potential misclassification/selection bias within the evidence set.

VA and early corneal status: There was no significant difference between MSICS and phacoemulsification in uncorrected distance VA at one week (OR 0.84; 95% CI 0.67-1.06; p=0.15; six studies; fixed-effect), and no significant difference in corneal oedema on day 1 (OR 0.90; 95% CI 0.70-1.16; p=0.42; six studies; fixed-effect).

Intraoperative complications: The only assessed complication was posterior capsule rupture. No significant difference was found between the two techniques (OR 1.07; 95% CI 0.73-1.58; p=0.72; five studies; fixed-effect).

Bias and sensitivity checks: Publication bias was assessed in the paper. The funnel plot appeared symmetric; the authors judged no publication bias detectable. As for the sensitivity analysis, excluding “low-quality” studies did not materially alter pooled effects.

AMSTAR assessment: The paper's strengths were that multiple databases were searched (including Chinese sources), study selection was performed in duplicate, and a funnel plot was used to assess publication bias, with sensitivity analyses also reported. However, the protocol was not registered, the inclusion/exclusion process did not appear to be fully transparent, Jadad scoring was used, there were inconsistent eligibility criteria (claimed RCT-only but included at least one non-RCT), and the meta-analyses predominantly used fixed-effects models despite clinical heterogeneity. Overall, it was of low-to-moderate quality, limited by methodological weaknesses and short follow-up in included trials.

Synthesis for this review: Within predominantly short-term follow-up (mostly ≤2-12 weeks), this review reports no statistically significant differences between MSICS and phacoemulsification for one-week UCVA, day-1 corneal oedema, or posterior capsule rupture. Findings are broadly in line with Reviews 1-3 on comparable safety and similar corrected vision; however, unlike those reviews, this analysis did not report pooled longer-term visual outcomes (e.g., BCVA at six to eight weeks/six months) or astigmatism advantages for phacoemulsification, and it mixes study designs despite RCT-only criteria, which limits confidence in its estimates.

Discussion

This overview of systematic reviews evaluated the comparative effectiveness and safety of phacoemulsification and MSICS. Across four included reviews, the findings were broadly consistent: both techniques provide excellent and comparable BCVA, while phacoemulsification offers modest advantages in UCVA and surgically induced astigmatism, and MSICS offers advantages in operative efficiency and cost.

Visual Outcomes

All reviews reported similar BCVA outcomes between techniques. The Cochrane review and Zhang et al. (RCT-only) [22,23] both showed no difference in achieving BCVA ≥6/18 or avoiding poor BCVA at six to eight weeks, with limited six-month data supporting the same conclusion. Gogate et al. and Zhang et al. [24, 25] found phacoemulsification to be associated with slightly better UCVA, particularly for thresholds above 6/9, possibly explained by lower induced astigmatism. However, these UCVA differences were modest, and their clinical importance may be context-dependent.

Although long-term BCVA outcomes are equivalent between techniques, small short-term UCVA advantages for phacoemulsification may be clinically meaningful. For patients, earlier unaided recovery of functional vision can translate into faster return to work and daily activities, which carry socioeconomic implications beyond visual outcomes alone.

Astigmatism and Corneal/Endothelial Outcomes

Phacoemulsification was consistently associated with lower surgically induced astigmatism, but this advantage did not translate into superior corrected vision. Endothelial cell loss was not significantly different between the two techniques in the pooled estimates. Corneal oedema was reported more frequently in the immediate postoperative period after phacoemulsification in the Cochrane review [22], though this was not consistent across all analyses.

Complications

Overall complication rates were similar between techniques. The Cochrane review and Zhang et al. [22,23] found no difference in posterior capsule rupture or other intraoperative complications. Gogate et al. [24] highlighted that during the surgical learning curve, MSICS may be safer, with fewer postoperative complications reported among trainees. Posterior capsule opacification was more common with MSICS in one RCT, although evidence remains limited, and follow-up durations were short in most studies.

Surgical Time and Cost

While MSICS is consistently less expensive in terms of operative costs, the broader economic impact is more complex. Earlier recovery of unaided functional vision with phacoemulsification could reduce indirect costs related to lost productivity and dependency. Future comparative studies incorporating both direct and indirect costs would help clarify the full economic implications of technique choice.

The relative efficiency of each technique also depends on the patient population and case mix. In many low-and middle-income countries where MSICS is commonly practised, patients often present later with advanced, denser cataracts. In these situations, MSICS can be performed more quickly and reliably than phacoemulsification, especially by expert surgeons trained in MSICS. Conversely, in high-income settings where patients typically present earlier with less advanced cataracts, phacoemulsification may be faster and more efficient. This context-specific variation highlights the importance of tailoring surgical choice not only to resources but also to patient demographics and cataract severity.

Methodological Quality of the Evidence

The evidence underpinning these reviews is limited by short follow-up, typically six to eight weeks, and in most cases not extending beyond six months. Trial methodological quality was variable: the Cochrane review [22] rated the overall certainty of evidence as low or very low, mainly due to risk of bias from unclear masking and attrition. Non-Cochrane reviews applied Jadad scoring, which is less comprehensive, and some (notably Ye et al. [25]) inconsistently applied their inclusion criteria by incorporating non-randomised studies. Publication bias was generally not detected, but the small number of included trials reduces confidence in these assessments.

Strengths of This Systematic Review

The strengths of this review include its comprehensive synthesis across multiple systematic reviews, allowing comparison of consistent and divergent findings. By examining both Cochrane and non-Cochrane reviews, this study provides a balanced perspective that incorporates differences in methodology, inclusion criteria, and regional emphasis. Another strength is the critical appraisal of risk of bias at the review level, highlighting where evidence is strongest (e.g., RCT-only analyses) and where methodological weaknesses limit confidence.

Weaknesses of This Systematic Review

This review also has limitations. First, it is reliant on the quality of the included systematic reviews, which themselves draw on a limited pool of primary trials. Most trials were conducted in South Asia, limiting generalisability to other populations and health systems. Second, there was considerable overlap of included trials across reviews, which restricts the amount of truly independent evidence available. Third, the included reviews varied in inclusion criteria (some mixed RCTs and observational studies), and definitions of outcomes were not always consistent, reducing comparability. Finally, this review is limited by the short follow-up durations of the underlying trials, meaning that long-term outcomes such as posterior capsule opacification, spectacle dependence, and patient-reported quality of life remain uncertain.

Consideration of Other Outcome Measures

Another limitation of the available evidence is that most systematic reviews focused narrowly on VA outcomes. Other clinically relevant metrics, such as contrast sensitivity, glare disability, and quality-of-life scores, were rarely, if ever, reported. Patient-reported experience and recovery times are also a factor, as mentioned previously. These functional and subjective measures may be more sensitive to differences between techniques, particularly in real-world settings where patient satisfaction and visual performance under varied conditions matter as much as visual acuity.

Implications for Practice

Taken together, the evidence supports both phacoemulsification and MSICS as safe and effective techniques. Phacoemulsification offers a modest optical advantage in the earlier stage of recovery, whereas MSICS provides clear benefits in cost and may be more appropriate in resource-constrained settings.

It should also be noted that in high-income countries, ophthalmology training predominantly focuses on phacoemulsification [27], meaning most surgeons are more proficient in this technique. This aligns with the typical patient populations in such settings, where cataracts often present earlier and are less advanced. In contrast, in many LMICs, patients frequently present later with mature or brunescent cataracts [28], for which MSICS may be the more efficient and practical option [29].

Choice of procedure should therefore balance patient expectation and preferences with system-level considerations, including cost-effectiveness and surgical training.

Implications for Research

Future trials should extend follow-up to assess long-term visual outcomes and quality of life and should recruit from diverse geographic settings beyond South Asia. Comparative cost-effectiveness analyses that incorporate real-world programmatic data are needed, particularly in the context of evolving phacoemulsification technology and training.

## Conclusions

To conclude, this systematic review of systematic reviews confirms that both phacoemulsification and MSICS provide comparable corrected visual outcomes and safety, with phacoemulsification offering modest UCVA and astigmatism benefits, and MSICS delivering faster, more affordable surgery. Strengths of this review lie in its comprehensive synthesis and risk-of-bias appraisal; its weaknesses reflect dependence on limited, overlapping, short-term evidence.

## Appendices

Appendix A

Search Strategy

**Table 3:** PubMed (253 studies)

Search	Query	Results
#6	Search: #5 NOT ("Animals"[Mesh] NOT "Humans"[Mesh])	253
#5	Search: #1 AND #2 AND #3 AND #4	253
#4	Search: "Visual Acuity"[MeSH] OR "visual acuit*"[tiab] OR "vision*"[tiab] OR "visual*"[tiab] OR UDVA[tiab] OR UDVA[tiab] OR CDVA[tiab] OR BCVA[tiab] OR UCVA[tiab] OR "LogMAR"[tiab]	9,87,668
#3	Search: "Cataract"[MeSH] OR cataract*[tiab] OR Pseudoaphakia[tiab] OR "lens opacit*"[tiab] OR "cataractous lens"[tiab]	77,814
#2	Search: "Small Incision Cataract Surger*"[tiab] OR "SICS"[tiab] OR MSICS[tiab]	1,143
#1	Search: "Phacoemulsification"[MeSH] OR Phacoemulsifi*[tiab] OR "phaco"[tiab] OR "lens emulsification"[tiab] OR "lenticular emulsion"[tiab] OR "phaco-emulsification"[tiab] OR "phako-emulsification"[tiab] OR "phakoemulsification"[tiab]	17,532

**Table 4:** Embase (346 studies) Embase: Excerpta Medica database

Search	Query	Results
#6	#5 NOT ([animals]/lim NOT [humans]/lim)	346
#5	#1 AND #2 AND #3 AND #4	346
#4	'visual acuity'/exp OR 'visual acuit*':ti,ab,kw OR 'vision*':ti,ab,kw OR 'visual*':ti,ab,kw OR 'udva':ti,ab,kw OR 'udva':ti,ab,kw OR 'cdva':ti,ab,kw OR 'bcva':ti,ab,kw OR 'ucva':ti,ab,kw OR 'logmar':ti,ab,kw	13,06,214
#3	'cataract'/exp OR 'cataract*':ti,ab,kw OR 'pseudoaphakia':ti,ab,kw OR 'lens opacit*':ti,ab,kw OR 'cataractous lens':ti,ab,kw	1,15,270
#2	'small incision cataract surger*':ti,ab,kw OR 'sics':ti,ab,kw OR 'msics':ti,ab,kw	1,612
#1	'phacoemulsification'/exp OR 'phacoemulsifi*':ti,ab,kw OR 'phaco':ti,ab,kw OR 'lens emulsification':ti,ab,kw OR 'lenticular emulsion':ti,ab,kw OR 'phaco-emulsification':ti,ab,kw OR 'phako-emulsification':ti,ab,kw OR 'phakoemulsification':ti,ab,kw	22,435

**Table 5:** Web of Science (476 studies)

Search	Query	Results
#5	#1 AND #2 AND #3 AND #4	476
#4	TS=('visual acuit*' OR 'vision*' OR 'visual*' OR 'udva' OR 'udva' OR 'cdva' OR 'bcva' OR 'ucva' OR 'logmar')	16,33,129
#3	TS=('cataract*' OR 'pseudoaphakia' OR 'lens opacit*' OR 'cataractous lens')	76,807
#2	TS=('small incision cataract surger*' OR 'sics' OR 'msics')	1,26,054
#1	TS=('phacoemulsifi*' OR 'phaco' OR 'lens emulsification' OR 'lenticular emulsion' OR 'phaco-emulsification' OR 'phako-emulsification' OR 'phakoemulsification')	14,505

**Table 6:** Cochrane (115 studies)

Search	Query	Results
#5	#1 AND #2 AND #3 AND #4	115
#4	(visual NEXT acuit* OR vision* OR visual* OR udva OR udva OR cdva OR bcva OR ucva OR logmar)	165840
#3	(cataract* OR pseudoaphakia OR lens NEXT opacit* OR cataractous NEXT lens)	10268
#2	(small NEXT incision NEXT cataract NEXT surger* OR sics OR msics)	376
#1	(phacoemulsifi* OR phaco OR lens NEXT emulsification OR lenticular NEXT emulsion OR phaco-emulsification OR phako-emulsification OR phakoemulsification)	3974
